# AirMLP: A Multilayer Perceptron Neural Network for Temporal Correction of PM2.5 Values in Turin

**DOI:** 10.3390/s23239446

**Published:** 2023-11-27

**Authors:** Martina Casari, Laura Po, Leonardo Zini

**Affiliations:** “Enzo Ferrari” Department of Engineering, University of Modena and Reggio Emilia, 41125 Modena, Italy; 258378@studenti.unimore.it

**Keywords:** air quality, neural network, low-cost sensors, laser-scattering technology, PM, multilayer perceptron

## Abstract

In recent times, pollution has emerged as a significant global concern, with European regulations stipulating limits on PM 2.5 particle levels. Addressing this challenge necessitates innovative approaches. Smart low-cost sensors suffer from imprecision, and can not replace legal stations in terms of accuracy, however, their potential to amplify the capillarity of air quality evaluation on the territory is not under discussion. In this paper, we propose an AI system to correct PM 2.5 levels in low-cost sensor data. Our research focuses on data from Turin, Italy, emphasizing the impact of humidity on low-cost sensor accuracy. In this study, different Neural Network architectures that vary the number of neurons per layer, consecutive records and batch sizes were used and compared to gain a deeper understanding of the network’s performance under various conditions. The AirMLP7-1500 model, with an impressive R-squared score of 0.932, stands out for its ability to correct PM 2.5 measurements. While our approach is tailored to the city of Turin, it offers a systematic methodology for the definition of those models and holds the promise to significantly improve the accuracy of air quality data collected from low-cost sensors, increasing the awareness of citizens and municipalities about this critical environmental information.

## 1. Introduction

In recent times, the issue of pollution has become a significant concern for humanity [1,2]. Exposure to particulate matter (PM) poses significant health risks, with strong associations established between PM exposure and cardiopulmonary morbidity and mortality, and lung cancer [3]. Fine particles, specifically those with a diameter of 2.5 μm or smaller, raise specific concerns because of their heightened toxicity, ability to penetrate the lungs deeply, and prolonged presence within the respiratory system [4,5]. Beyond health impacts, PM 2.5 also contributes to environmental problems such as reduced visibility, ecological damage such as soil nutrient depletion and acid rain effects, as well as material deterioration, exemplified by the discolouration of cultural landmarks [6,7,8]. In response to this mounting concern, European regulations and U.S. National Ambient Air Quality Standards (NAAQS) [9,10] have implemented stringent measures. These actions involve the establishment of rigorous standards that limit the number of days in a year when PM 2.5 and 10 levels can exceed defined thresholds. Furthermore, they have enforced restrictions on particulate matter emissions from a wide range of sources in various industries.

The concentrations of PM exhibit inherent spatial and temporal variations, leading to differing personal exposure levels [11]. To tackle this challenge and expand the monitored areas, various initiatives have been undertaken to create affordable PM sensor networks [12,13,14,15]. Usually, these networks employ low-cost sensors that offer advantages such as scalability, real-time data, and citizen science participation; while these widely distributed sensors cannot completely replace reference stations in terms of accuracy, they play a vital role in advancing air quality research [16,17,18].

There are various types of low-cost (LC) sensors designed for measuring pollutants, broadly classified into gases and particulate matter [19,20,21]. In the context of our paper, we focus on PM low-cost sensors that employ laser scattering techniques to estimate particle size and number concentrations, which are subsequently converted into mass concentrations using proprietary algorithms.

Laser scattering methods have limitations in accuracy compared to more expensive reference instruments. Moreover, low-cost sensors may be sensitive to environmental factors such as humidity, temperature, and atmospheric pressure, which can influence sensor performance and be susceptible to interference from other pollutants or sources, leading to erroneous readings. Artificial intelligence methods have demonstrated their effectiveness in enhancing the precision of low-cost sensor measurements. Many of these approaches involve a calibration procedure that requires positioning the low-cost sensors close to reference stations [22,23] and then aligning the data exploiting machine-learning techniques or neural networks.

In this paper, we address the issue of a calibration of low-cost PM sensors using Multilayer Perceptron (MLP) networks, which have a track record of outperforming other algorithms in similar scenarios, as highlighted in [24]. Our objective is to establish a basis for future research initiatives, starting with Turin’s city, Italy. We aim to introduce a systematic methodology applicable to training neural networks in diverse settings beyond our initial study area.

Our research encompasses several key components:

1. Data Understanding and Assembling: Our first step involves the assembly of a comprehensive dataset. We collect data from multiple sources, including five low-cost sensors and one reference station. The resulting dataset is designed to capture the temporal patterns inherent in the data and to consider the influence of various meteorological factors on the detected concentrations;
2. Neural Network Training: We train various neural network models, using the assembled dataset. These models are designed to minimise the error introduced by the low-cost sensor in estimating PM 2.5 concentrations. Different hyperparameters to identify the optimal model configuration were explored and compared.
3. Results Comparison: A comparative analysis of the best-performing model has been conducted, and additional insights and considerations derived from our findings are provided.

The rest of the paper is organised as follows. In Section 2, we describe the devices used in the study, elucidate the process of dataset creation, and introduce the MLP architectures employed. Section 3 delves into the tests carried out and provides an analysis of the results achieved through a comparison of different architectures. Section 4 and Section 5 are dedicated to the discussion and conclusions, respectively, drawing upon our discoveries and providing valuable insights for potential avenues of future research.

## 2. Materials and Methods

This section delves into the air quality sensor characteristics employed in the study and the main challenges related to their deployment, the creation of a dataset designed to capture the temporal nature of the sensor data and the neural network architecture used in our research.

### 2.1. Air Quality Sensors

In the context of our research, we have access to observations obtained from five low-cost devices, named Arianna and produced by Wiseair [25], located in Turin; positioned near a Tecora Sequential Unit, a reference station that allows the automated and sequential collections of the atmospherical PM; and installed and managed by Arpa Piemonte [26,27]. These devices were placed in a green area, distanced from the most congested streets. The dataset covers four months, with three devices deployed for two months between March and April and the remaining two devices collecting data for two months from October to November.

Each Arianna device includes an SPS30 sensor [28,29] and sensors for measuring relative humidity (RH) and temperature. SPS30 employs laser scattering technology (see Section 2.1.1) to measure PM 1, PM 2.5, PM 4, and PM 10 concentrations, giving in output both number and mass concentration. Unfortunately, the number concentration was not available at the time of this study. Specifications relative to PM 2.5 are reported in Table 1.

In summary, this study incorporates a range of features, including PM 2.5 concentrations (μg/m${}^{3}$), RH and temperature (%), and temperature (${}^{\circ }$C), and integrates these with additional meteorological factors such as atmospheric pressure (hPa), cloud coverage (%), and wind speed (m/s). These features are the most significant in the context of air quality monitoring. Pressure affects air density, which, in turn, impacts the dispersion of particulate matter in the air. Cloud coverage is related to RH [30]. Temperature plays a main role in influencing RH, which is a major concern in this study. Wind speed affects the dispersion of particles. All of these factors have the potential to influence the state of air quality over time.

An important aspect to consider is that when Arpa station records raw data below the detection limit, the result is assumed to be equal to half of the detection limit. This approach is referred to as a *medium bound* technique (when the concentration of a target substance is below the detection limit, it is typically considered to be estimated as half of the detection limit). As a result, the Agency introduces a lower limit in the ground truth data, which is set at 4 μg/m${}^{3}$ in this case.

#### 2.1.1. Laser-Scattering Technology

**Figure 1:**
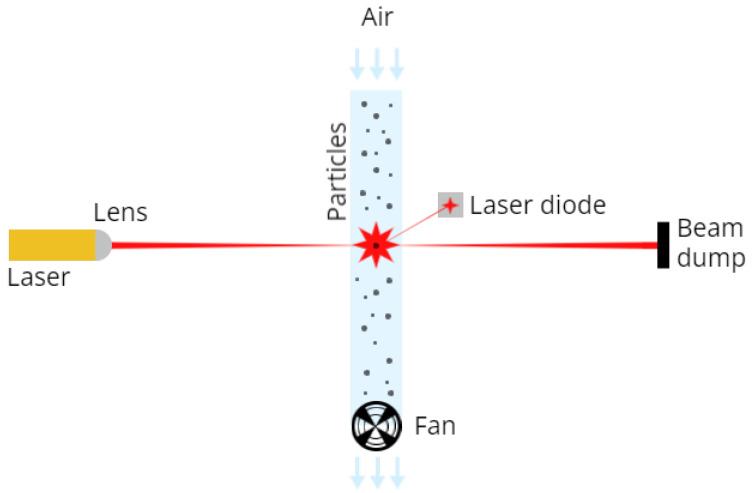
Simple diagram of laser scattering technology.

In the realm of air quality monitoring, various types of low-cost sensors have been employed to measure pollutants, particularly PM. We categorise the instruments used as optical particle sensors (OPS), which can be further classified into two primary types: nephelometers and optical particle counters (OPC) [31]. Nephelometers operate by measuring particles collectively, capturing light scattered by all particles across a wide range of angles. In contrast, OPC detects particles individually. A simple laser scattering example is given in Figure 1. From the light scattered, by applying the Mie theory, it is possible to calculate the equivalent particle diameter and the number of particles with different diameters per unit volume. The data collected are then converted into particle mass concentration, expressed in units of micrograms per cubic meter (μg/m${}^{3}$).

The term low-cost sensor encompasses a wide range of technologies, ranging from sensors that cost tens of dollars to those with higher price tags, sometimes reaching a few thousand dollars. In contrast, reference stations are significantly more expensive, often exceeding tens of thousands of dollars in cost.

Current state-of-the-art research primarily focuses on data collected from sensors priced below a few hundred dollars. These sensors are affordable for citizens, making them accessible, but they also come with a level of simplicity. The straightforward technology used in these sensors presents challenges in accurately detecting PM concentrations.

Table 2 compares various low-cost PM sensors frequently discussed in the literature [31,32,33,34,35,36,37]. These sensors are recognised for their simplicity and affordability but lack additional technology to counteract the impact of external factors, including meteorological conditions.

In contrast, Table 3 highlights devices that combine low-cost sensors with supplementary technologies. For instance, the device employed in this study, named Arianna, features an SPS30 sensor, RH and temperature sensors, and a filter to exclude larger external objects, such as insects, from entering the device. It is worth noting that Arianna does not include an air conditioning system, as it relies on solar panels for power, which limits its energy resources. In contrast, the other devices in Table 3 use such technology to reduce humidity, a critical factor that will be discussed further in Section 2.1.2.

Although the inclusion of a drying system is essential for accuracy, it increases the cost, making these devices less accessible to the general public. In fact, as shown in Table 2, low-cost sensors are remarkably affordable. In contrast, the devices presented in Table 3 are significantly more expensive, often exceeding thousands of dollars due to their heightened complexity. The cost of these devices may vary depending on factors such as the quantity sold and whether they are available for purchase or exclusively for rental.

#### 2.1.2. Hygroscopicity Issue

One significant challenge faced by these low-cost sensors is their susceptibility to various influencing factors, particularly the impact of humidity [46,47].

**Figure 2:**
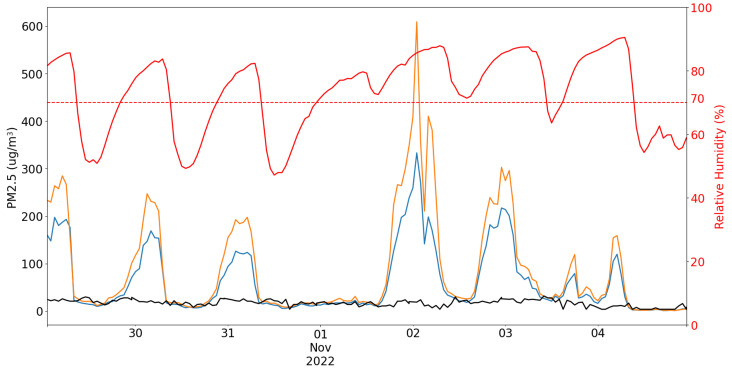
Comparison of PM levels measured by two low-cost sensors (represented by blue and orange lines) with data from the Arpa reference station (black line), accompanied by RH measurements (red) and an RH threshold of 70% (dotted red line).

When humidity levels increase, the low-cost sensor reports higher PM 2.5 values than the actual concentration. This stems from the hygroscopic nature of airborne particles [48,49]. As illustrated in Figure 2, when RH, indicated by the red line, exceeds a certain threshold, there is a noticeable increase in the reported PM 2.5 values, represented by the blue and orange lines. This increase in PM 2.5 concentration, as observed in the low-cost sensor data, deviates significantly from the measurements obtained from the reference station, shown in black. This discrepancy arises from the humidity being attracted and held via either absorption or adsorption from the surrounding environment, leading to particle size expansion. Consequently, the low-cost sensor detects an elevated number of PM 2.5 particles, resulting in inaccurate measurements.

Simply eliminating or truncating these humidity-induced spikes is an inadequate solution, particularly in areas with elevated humidity levels, as it would lead to a substantial loss of data. When referring to *elevated humidity levels*, we mean RH exceeding 70% [48]. However, it is essential to emphasise that this threshold may vary based on the specific sensor in use. Experiments have demonstrated that low-cost devices equipped with dryers at their inlets measured an average of 64% lower PM 2.5 concentrations with elevated humidity levels compared to sensors without these dryers [50]. This underscores the critical importance of mitigating fluctuations in sensor readings induced by humidity [51]. However, it is important to note that the addition of a dryer increases the cost of the sensor and may not always be a feasible solution (see Table 2 and Table 3).

More comprehensive approaches involve calibration or mitigation of the hygroscopicity effect, which involves developing models capable of effectively addressing this issue while potentially addressing other external factors contributing to erratic sensor readings.

#### 2.1.3. Calibration Challenges

Previous research has extensively explored the influence of variables such as RH and temperature on sensor performance [46,52,53]. These studies have underscored the pressing need for correction models to rectify inaccuracies in sensor data induced by humidity.

Nevertheless, it is important to be aware that calibration challenges may arise when models are trained in one location but are later deployed in different places [54]. This can be attributed to variations in pollutant sources and environmental contexts, which can result in significant spatiotemporal differences. To tackle this issue, methods like corrective functions have been proposed [11,55,56]. Corrective functions are particularly valuable when only humidity and PM data are available. They derive a correction coefficient based on humidity levels to reduce detected PM concentrations. While they can be fine-tuned by calibrating against a reference station, this step is not always mandatory. Alternatively, an approach involves optimizing function parameters minimizing the correlation between humidity and PM concentration.

Another challenge includes the need for sensor maintenance, typically involving recalibration approximately twice a year [57]. Additionally, many of these techniques address mass concentration, aligning with the focus of our study. However, it is worth noting that directly considerate particle counts could offer advantages [58,59], as it sidesteps potential complexities associated with the conversion from counts to mass, and because a larger particle size translates into a shift of particles into a smaller diameter bin and not in a real diminishing of particle mass. Data frequency is another factor to consider, as reference stations often provide data daily or hourly, limiting the potential for real-time monitoring and fine-grain frequency accuracy.

Our study leverages neural networks as superior models for improving the accuracy of PM concentrations [24], particularly for sensor data affected by the hygroscopicity issue in high humidity regions (see Section 2.1.2). In these conditions, traditional linear correction methods often prove inadequate. In contrast to simpler methodologies, however, MLPs introduce certain limitations. These encompass reduced interpretability of the model’s decision-making process, potentially constraining their utility in quantitative applications. Furthermore, the intricacies associated with model design and the meticulous fine-tuning of hyperparameters contribute to the challenges posed by this approach.

Our research aims to develop a systematic methodology for future research in this field. However, like other calibration techniques, this approach is dependent on the specific location for which it is trained. To address this local aspect, a potential solution could involve collecting data from the same sensor model deployed in different locations. Currently, this poses a challenge due to the scarcity of resources for co-locating low-cost sensors with reference stations.

### 2.2. Dataset Assembly

**Figure 3:**
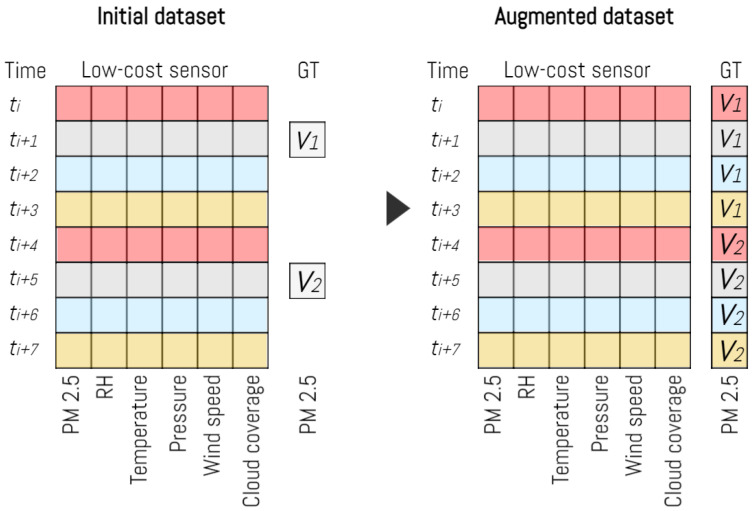
Dataset augmentation phase.

To handle the dissimilarity in data frequencies between the low-cost devices (generating four data entries per hour) and the reference station (providing one data entry per hour), we implemented a data augmentation strategy. Instead of averaging the low-cost device data on an hourly basis, and downsizing the dataset, we opted for upsizing the dataset, as reported in Figure 3. We replicated the ground truth values for each input vector originating from the low-cost devices based on the timestamp proximity.

This approach allows you to maintain a consistent and synchronised dataset, even though the low-cost devices generate data more frequently than the reference station. By generating four corresponding input vectors for each hour of data collected by the reference station, we ensure that the data from the low-cost devices is still represented accurately in relation to the hourly measurements from the reference station.

This technique can be useful when working with data sources with varying sampling frequencies and can help ensure that all data sources are comparable and synchronised for further analysis or modelling.

As previously mentioned, each data record from low-cost devices comprised six distinct features (PM 2.5, RH, temperature, pressure, wind speed, and cloud coverage), and a timestamp indicating the temporal aspect.

**Figure 4:**
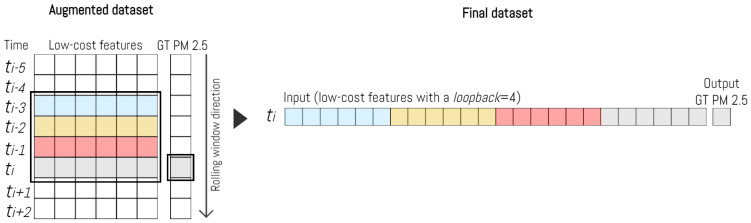
Dataset construction schema with a *loopback* of 4.

To improve our ability to account for the temporal context, we developed a dataset structure that connects multiple consecutive historical records, which we refer to as a *loopback*, associated with the same device, as illustrated in Figure 4. The ground truth value for each input vector was determined based on the PM 2.5 reading from Arpa corresponding to the final observation within the interval. This process was systematically executed for every entry in the dataset.

We consistently applied this approach to all the devices under investigation and integrated the results into a unified dataset. As a result, each entry was enriched with valuable temporal insights.

Then, the dataset was partitioned into training (75%) and test sets (25%), and a shuffling process was applied to the data during this segmentation. It is worth noting that a sensible precaution, which was not integrated into this research, would involve taking into account the data’s temporal characteristics to prevent closely related data points from ending up in both the training and test sets.

The dataset in our study remains unprocessed, and no additional steps have been taken. It is worth mentioning that potential future steps could involve outlier removal, for which we may utilise techniques described in [60,61]. Furthermore, the dataset has not been normalised, and it is noteworthy that, in our results, normalization led to inferior performance.

### 2.3. MLP Architectures

Our approach involves a systematic exploration of various neural network architectures, each characterised by a unique set of hyperparameters. The primary evaluation metric for their performance is the coefficient of determination, denoted as $R^{2}$.

All these architectures have a consistent input size, which is six times the length of the *loopback*, as there are six features involved. They generate a single neuron output, given the regression nature of the task. The architectural diversity primarily revolves around variations in depth and width.

We conducted experiments using architectures with depths ranging from six to eight layers, maintaining uniform layer widths throughout. Each architecture consisted of a linear layer followed by a Rectified Linear Unit (ReLU) activation function.

In the training process, we employed the L1 Error Loss. Additionally, we introduced a variant of the architecture in which the last hidden layer contained half the number of neurons, specifically when working with seven or eight layers. In total, we considered five distinct architectures, collectively referred to as AirMLP. The nomenclature is based on the layer count, the presence of an “h” denoting half neurons in the last layer, and the number of neurons per layer. For example, an architecture with seven layers and 500 neurons per layer is labelled as AirMLP7-500.

Furthermore, we investigated the potential advantages of the dataset normalization and the inclusion of a batch normalization layer, which normalises data across batches while learning affine parameters. Our experimentation revealed significant findings, indicating that the inclusion of a batch normalization layer, without prior dataset normalization, yielded superior results.

No other regularization techniques were applied.

## 3. Results

Since we utilised multilayer perceptron architectures, a key consideration was finding the right network size that achieves a harmonious blend of excellent performance, preventing overfitting, and handling computational demands. This prompted us to investigate a range of architectures with different combinations of hyperparameters.

We established a systematic and comprehensive pipeline that facilitated the training of each network with a unique combination of hyperparameters. Specifically, we focused on two key hyperparameters: the number of consecutive old records used to create the new dataset, the *loopback*, and the number of neurons per layer. These parameters collectively defined the specific architecture of the model being trained. Furthermore, we conducted experiments using three different batch sizes to assess their impact during training.

The hyperparameter values under consideration were as follows: the number of neurons per layer spanned from [300, 500, 700], the number of consecutive records fluctuated between [6, 12, 20], and batch sizes covered [64, 256, 512]. We assessed these hyperparameter combinations across five distinct architectures, resulting in a total of 135 potential configurations, each requiring training and evaluation. This thorough method enabled us to acquire a more profound insight into the network’s performance across a range of conditions.

In Table 4, the R^2^ scores corresponding to each architecture evaluated on the test set, alongside different sets of hyperparameters, is provided. Each column in the table represents the results obtained for various combinations of batch size and consecutive records, with the column name structured as *batch size : loopback number*. It is important to note that the values presented are not averages of multiple test results; instead, they represent a single measurement for each configuration.

The data in the table highlights some noteworthy trends. First and foremost, increasing the *loopback* consistently results in better performance. This is a valuable insight as it suggests that capturing longer-term temporal dependencies enhances the network’s ability to correct PM 2.5 values. Conversely, increasing the batch size tends to lead to a decrease in the $R^{2}$ value. This implies that smaller batch sizes might be more effective in training the neural network.

Furthermore, the number of neurons per layer and the depth of the network, indicated by the number of layers, impacts performance. Increasing the number of neurons per layer within the network often results in better $R^{2}$ values. Keeping a constant number of neurons, deeper networks tend to yield higher $R^{2}$ values. This suggests that having a more complex model with a higher capacity for learning intricate patterns is beneficial.

As highlighted in the results, the optimal configuration for each model consists of a higher number of neurons per layer and a batch size of 64. However, to gain a more comprehensive understanding, we should investigate the effects of further increasing the number of neurons per layer and assess whether this leads to the onset of overfitting. This analysis will help us strike a balance between model complexity and performance, ensuring that we do not compromise generalization capabilities while seeking optimal performance.

**Figure 5:**
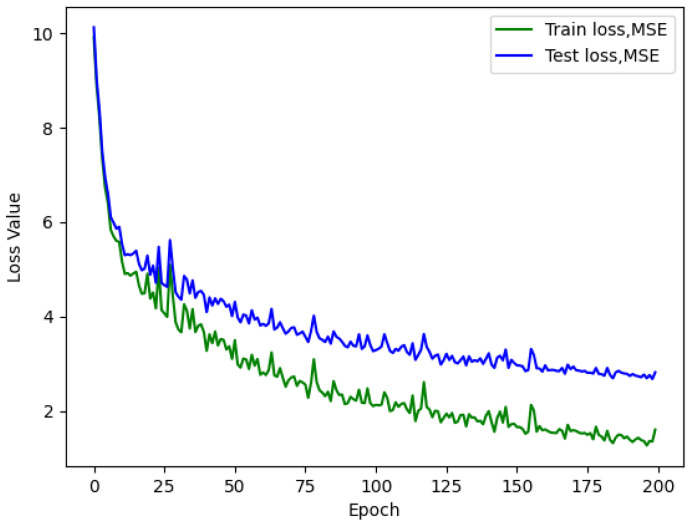
AirMLP8-700 loss with a batch size of 64, test L1 value of 2.732.

**Figure 6:**
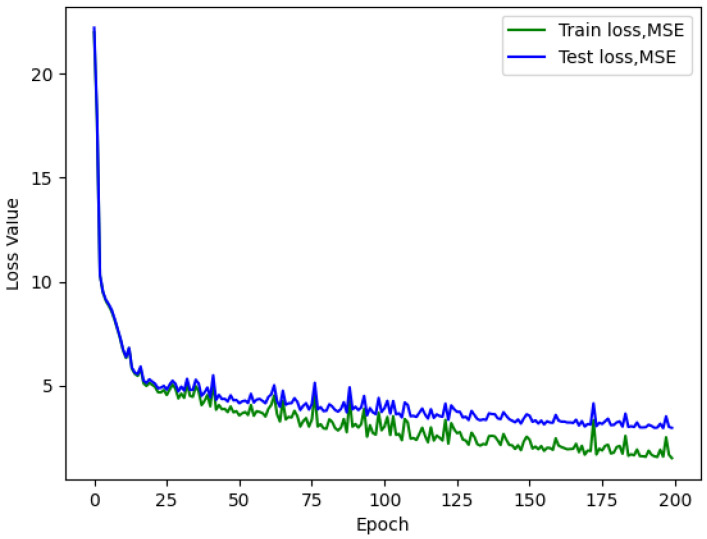
AirMLP8-700 loss with a batch size of 256, test L1 value of 2.976.

**Figure 7:**
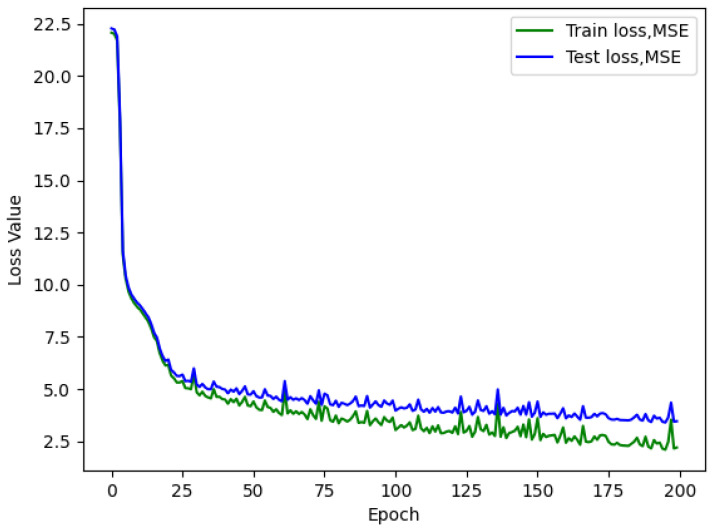
AirMLP8-700 loss with a batch size of 512; test L1 value of 3.461.

Analyzing the loss graph reveals several valuable insights. Firstly, during the initial training attempts, it becomes evident that overfitting is not a prevalent issue, as demonstrated in Figure 5, Figure 6 and Figure 7. This observation suggests that there is room to consider increasing the network size without immediate concerns about overfitting.

Furthermore, the loss graph unveils two significant insights. When a larger batch size is employed, the network converges more rapidly and smoothly, but at the cost of a higher loss value, ultimately leading to poorer performance. Conversely, when a smaller batch size is used, the network achieves convergence at a lower loss value, even with an identical number of neurons.

The continuous descent of the loss curves implies that there is potential for further enhancement in the model’s performance. Rather than employing a fixed number of epochs for training, as demonstrated in this study, the implementation of an early stopping technique warrants consideration and could yield substantial benefits.

Following our initial assessment, which identified the most effective hyperparameter set for each model as a batch size of 64, a *loopback* of 20 consecutive records (indicating the inclusion of 19 past observations in addition to the current one being corrected), and 700 neurons per layer, it becomes imperative to investigate the potential benefits of further increasing the number of neurons per layer.

In pursuit of this objective, we carried out additional training sessions for all five models, using varying neuron counts of 900, 1100, and 1500 per layer, while maintaining a consistent 20 consecutive records and a batch size of 64. As depicted in Table 5, it is evident that the model achieves optimal performance with seven layers and 1500 neurons in each layer. This observation holds for different neuron counts as well, signifying that employing seven layers seems adequate for representing this regression task effectively.

**Figure 8:**
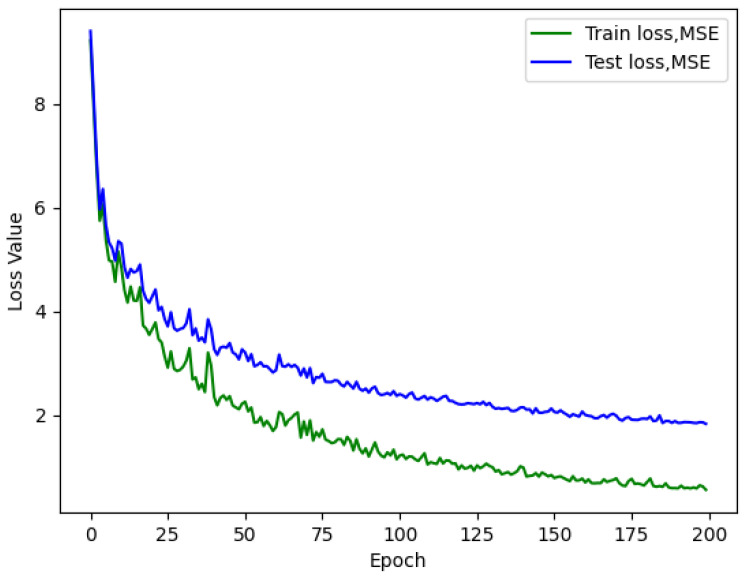
AirMLP7-1500 loss. Batch size of 64 and a *loopback* of 20.

The outcomes depicted in Figure 8 are rather remarkable, as they reveal not only enhancements in terms of the R^2^ metrics but also significant improvements in the loss reduction process during training. The loss curves now exhibit a smoother descent, and they converge to lower values compared to the previous configurations. These findings strongly indicate that augmenting the number of neurons per layer has exerted a positive influence on both the model’s predictive performance and its training stability.

Upon inspecting the figures, it becomes evident that there is still no indication of overfitting throughout the training process. Notably, the AirMLP7-1500 model performs well, potentially serving as a solid foundation for further exploration.

While it is tempting to increase the network’s dimensions even further, it is imperative to carefully weigh the trade-off between potential performance enhancements, which may be modest, and the accompanying escalation in computational requirements.

**Figure 9:**
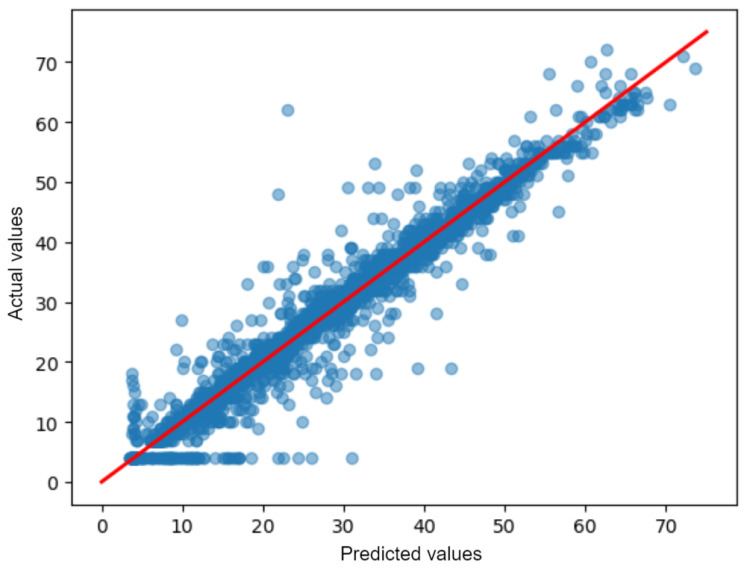
Comparison between predicted results and actual values from the AirMLP7-1500 model on the test dataset (blue dots).

Evaluating the AirMLP7-1500 model’s performance on the test dataset involves contrasting its predicted results with the actual values. To highlight this, we drew the predicted test scatter plot in Figure 9. The ideal scenario is when these predicted values (blue dots) perfectly align along the diagonal line (the red line in Figure 9), indicating that the model’s predictions match the reference station’s actual values. Any deviations from this diagonal line signify, instead, the magnitude of prediction errors and provide insights into the model’s performance. The Figure shows a strong correlation between the predicted values generated by the model and the original values (ground truth); indeed, the predicted values are closely clustered around the diagonal line. This suggests that the neural network AirMLP7-1500 is effective in accurately predicting PM 2.5 concentrations.

The figure also shows a negative consequence of the lower threshold applied to the data by the Arpa Agency (described in Section 2.1) is visible in the left-bottom part of the plot. The data equal to such threshold are horizontally aligned at y=4, and the NN reproduce this threshold, visible at x=4.

**Figure 10:**
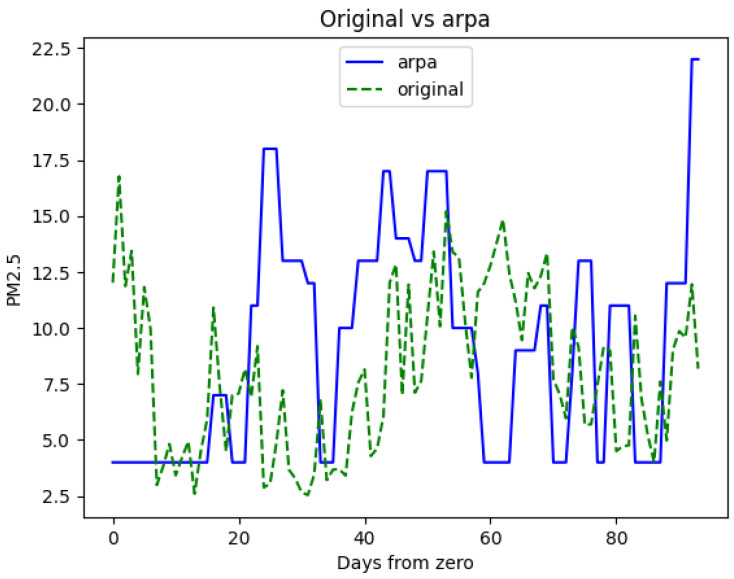
Original vs. Arpa ground truth data.

**Figure 11:**
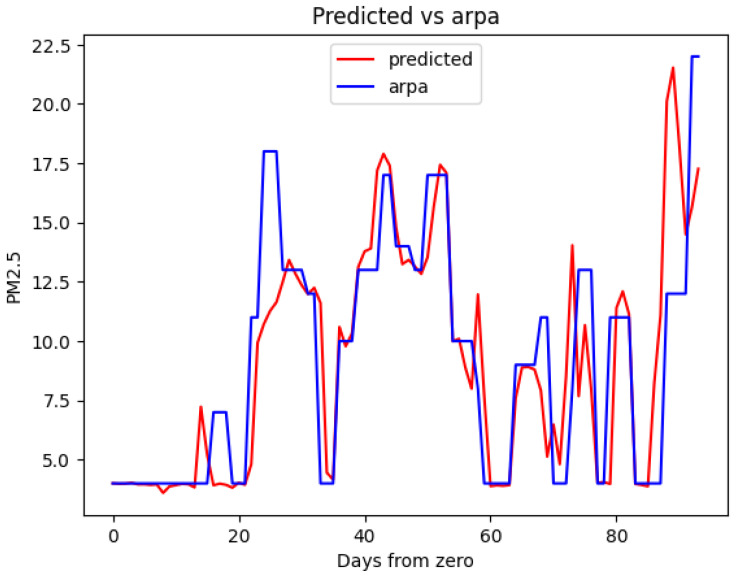
AirMLP7-1500’s predicted output vs. Arpa ground truth data.

Now, let us examine a secondary test dataset comprising consecutive data from a few days, which is not part of the training set. The PM 2.5 values originally recorded and the PM 2.5 values generated by the AirMLP7-1500 model are contrasted with the ground truth (Arpa), as illustrated in Figure 10 and Figure 11, respectively. This analysis will offer insights into the model’s ability to adapt to novel, unobserved days and its performance in a real-world context beyond the training data.

The first thing you may notice is the notable improvement in sensor data alignment compared to Arpa when introducing the predictions from the AirMLP7-1500 model. Moreover, the predictions do not exhibit any unusual behaviour, indicating a smooth correction of the data.

Some minor observations are that in both Figure 10 and Figure 11, the Arpa line displays plateaus due to the replication of data to match the 15-min low-cost granularity frequency, and in Figure 11, there are instances where the model does not precisely reconstruct certain peaks. For instance, around day 25, the neural network entirely misses a peak, and near day 90, it anticipates one. These discrepancies may be attributed to the data augmentation method employed, which might lack access to the exact PM concentration values.

A particular aspect that can be noticed is the lower threshold imposed by Arpa through the *medium bound* technique, as discussed in Section 2.1, which is learned by the neural network. This lower threshold is visible in Figure 10 and Figure 11 in the line representing Arpa. It does not reach a PM concentration of 0 but is imposed at 4 μg/m${}^{3}$.

These outcomes are indicators of the model’s generalization capabilities and provide further evidence that the network does not suffer from overfitting. They highlight the robustness of the model and its ability to provide accurate PM 2.5 estimates, reinforcing its potential for real-world applications in air quality monitoring.

## 4. Discussion

In this Section, we explore significant discoveries, potential implications, and future directions.

The results, provided in Section 3, underscored the capability of MLPs to effectively represent and address the problem. These networks yield commendable $R^{2}$ scores. It is important to note that overfitting is not observed in this context, affirming the robustness of the models. Furthermore, the batch size significantly influenced performance, with larger batch sizes leading to a decrease in performance. On the other hand, employing a wider network with 1500 neurons per layer proved to be the optimal choice. Additionally, networks with seven or eight layers demonstrated a better performance. The inclusion of a *loopback* mechanism, allowing the network to consider a longer history of data, proved to be advantageous. The performance results indicate that leveraging more historical data leads to improved model accuracy.

Regarding the influence of RH, our study emphasizes the significant impact of humidity on the accuracy of low-cost PM 2.5 measurements. When RH surpasses a threshold of 70%, low-cost sensors tend to overestimate PM 2.5 levels. This distortion can undermine the reliability of air quality assessments, highlighting the limitations of such data in pollution-related decision-making.

Among the models, the AirMLP7-1500 model stood out, achieving an impressive R^2^ score of 0.932. The achieved R^2^ result aligns with the performance of neural networks reported in the existing literature, which typically surpasses 0.9 [24]. This again underscores the potential for AI-based correction methods to enhance the accuracy of low-cost sensor data.

The proposed method is not without limitations, particularly in terms of dataset shuffling and augmentation techniques. The specular pattern between training loss and test loss is shown in all the loss figures. This phenomenon could likely be attributed to the initial shuffling of data, which may not have adequately accounted for the presence of very similar data points in both the training and test sets. Further investigation and refinement of the data splitting and shuffling procedures could potentially mitigate this issue and provide a more accurate representation of the model’s performance on unseen data.

Additionally, in the context of handling Arpa reference station data, rather than the current approach of oversampling and repeating values to achieve finer granularity from 1 h to 15 min frequency, it is crucial to explore alternative strategies. One such strategy involves developing a weighted mean between the value at a given time point and the subsequent hour’s value. This exploratory approach holds the potential to not only enhance the quality of the ground truth data but also mitigate the erroneous reconstruction of peaks.

An important limitation to acknowledge is that the proposed correction models are tailored to a specific location and a particular set of low-cost devices. As previously highlighted, applying these models to different sensors or locations may yield sub-optimal results.

Numerous promising avenues for future research emerge from this study. One immediate direction is to investigate the impact of further increasing the model’s complexity, with a focus on balancing improved performance with computational resources. Regularization techniques like dropout could also be explored to enhance model robustness.

Considering seasonality when training PM 2.5 correction models is another promising direction. Seasonal variations in air quality are a well-known phenomenon [62,63]. By training separate models tailored to different seasons, it is possible to capture and correct seasonal variations in PM 2.5 measurements. Moreover, incorporating temporal information into the dataset is a strategy that can further enhance the network’s ability to differentiate between diverse environmental contexts. This could involve including features such as day of the week, time of day, or even specific holidays or events that might impact air quality. By accounting for both seasonality and temporal dynamics, the models can become more adaptive and provide accurate corrections across various environmental scenarios. This approach aligns with the idea of developing context-aware models that tailor their corrections based on the prevailing conditions [64], ultimately advancing the effectiveness of low-cost sensor data in air quality monitoring.

In addition, extending the models’ evaluation to new, unseen data from diverse geographical locations is essential to assess their generalization capabilities [65]. This would help determine the models’ adaptability to varying environmental conditions. Incorporating data from low-cost laser scattering sensors manufactured by different companies is also an important consideration. Different sensor models may exhibit unique characteristics and behaviours. Testing the models with data from various sensor manufacturers can help validate their robustness and ensure that they perform effectively across a spectrum of sensor types.

Beyond correction, the models developed here could be adapted for anomaly detection, helping to identify unusual PM 2.5 readings that may indicate pollution events or sensor malfunctions. Additionally, transitioning from correction to prediction could enable the forecasting of future PM 2.5 levels based on atmospheric conditions, contributing to proactive pollution management at the installation site.

Overall, the findings of this research provide valuable insights into the potential of neural networks, specifically MLPs, in improving the accuracy of low-cost sensor measurements. The study’s results contribute to the broader understanding of machine learning techniques in air quality monitoring and provide a foundation for further exploration and application in this field.

## 5. Conclusions

In this paper, we have addressed a critical challenge, which is improving the accuracy of PM 2.5 measurements acquired from low-cost devices equipped with an SPS30 sensor using MLP networks.

Our research employed data collected from five distinct low-cost devices for five months. These devices were located near a Tecora Arpa reference station in Turin. The data underwent augmentation to align with the low-cost device granularity, and it was provided to the neural networks in its raw form.

Remarkably, the AirMLP7-1500 model, which consists of seven layers, each containing 1500 neurons, and includes a *loopback* of 20, achieved an impressive R^2^ score of 0.932, effectively mitigating the impact of hygroscopicity.

The significance of this study provides the foundation for a systematic methodology that can be adapted for training similar models in various environmental contexts. This level of customization is a key strength, as it enables the model to effectively capture the complex relationships between local atmospheric parameters and the accurate detection of PM 2.5 concentration levels.

Looking ahead, our work opens the door to further investigations. These avenues include exploring the impact of increasing network complexity while guarding against overfitting, the application of regularization techniques, and enhanced data preprocessing before the training phase. Considerations such as seasonality, improved data preprocessing, and extending the applicability of the models to various geographical locations and sensor types are also promising directions for future research.

We argue that this study serves as a solid foundation for broader and more versatile inquiries within the realm of air quality monitoring and correction, setting the stage for further advancements in this critical field.

## Figures and Tables

**Table 1:** SPS30 specifications related to PM 2.5.

Parameter	Conditions	Value	Units
Mass concentration range	-	0 to 1000	μg/m${}^{3}$
Mass concentration size range	PM 2.5	0.3 to 2.5	μm
Mass concentration precision for PM 2.5	0 to 100 μg/m${}^{3}$	±10	μg/m${}^{3}$
	100 to 1000 μg/m${}^{3}$	±10	% m.v. *
Maximum long-term mass concentration precision limit drift	0 to 100 μg/m${}^{3}$	±1.25	μg/m${}^{3}$/year
	100 to 1000 μg/m${}^{3}$	±1.25	% m.v./year
Number concentration range	-	0 to 3000	#/cm${}^{3}$
Number concentration size range	PM 2.5	0.3 to 2.5	μm
Number concentration precision for PM 2.5	0 to 1000 #/cm${}^{3}$	±100	#/cm${}^{3}$
	1000 to 3000 #/cm${}^{3}$	±10	% m.v.
Maximum long-term number concentration precision limit drift	0 to 1000 #/cm${}^{3}$	±12.5	#/cm${}^{3}$/year
	1000 to 3000 #/cm${}^{3}$	±1.25	% m.v./year
Lifetime	24 h/day operating	>10	years
Temperature range	-	10 to 40	${}^{\circ }$C
Relative humidity	-	20 to 80	%

* % m.v. means “% of the measured value”.

**Table 2:** A comparison of PM low-cost laser-scattering sensors.

Model	Make	Technology	PM Detected	Output	Approximate Cost (USD)
SDS011 [38]	Nova	Laser scattering OPC	PM 2.5, PM 10	Particle mass concentration	30
SPS30 [39]	Sensirion	Laser scattering OPC	PM 1, PM 2.5, PM 4, PM 10	Particle count and mass concentration	50
HPMA115C0	Honeywell	Laser-based light scattering	PM 1, PM 2.5, PM 4, PM 10	Particle mass concentration	80
HPMA115S0 [40]			PM 2.5, PM 10		
OPC-N2/OPC-N3 [41]	Alphasense	Laser scattering OPC	PM 1, PM 2.5, PM 10	Particle mass concentration	500

**Table 3:** A comparison of PM devices.

Model	Make	Air Conditioner or Built-in Heater	Technology	PM Detected	Output
Arianna	Wiseair [25]	No	Laser scattering OPC	PM 1, PM 2.5, PM 4, PM 10	Fine particle counts and mass concentration
10,000/12,000 [42,43]	Particle Plus	Yes (humidity and condensation control)	Optical light scattering	PM 0.3, PM 0.5, PM 1, PM 2.5, PM 5, PM 10	Fine particle counts and mass concentration
AM520 [44]	SidePak	Yes (Inlet conditioner)	Light-scattering laser photometers	PM 0.8, PM 1, PM 2.5, PM 4, PM 10	Particle mass concentration
AQMesh [45]	Environmental Instruments	Yes	Light-scattering OPC	PM 1, PM 2.5, PM 10	Particle mass concentration

**Table 4:** R^2^ score on models trained differently. The meaning of the first row is [batch size]:[num records].

Model	Neurons	64:6	256:6	512:6	64:12	256:12	512:12	64:20	256:20	512:20
AirMLP6	300	0.801	0.772	0.752	0.827	0.793	0.758	0.859	0.812	0.788
	500	0.833	0.801	0.769	0.863	0.830	0.808	0.884	0.841	0.821
	700	0.862	0.821	0.780	0.878	0.853	0.819	**0.901**	0.858	0.849
AirMLP7	300	0.811	0.780	0.760	0.847	0.805	0.778	0.865	0.823	0.801
	500	0.844	0.814	0.770	0.883	0.838	0.815	0.888	0.859	0.826
	700	0.857	0.826	0.813	0.886	0.855	0.841	**0.904**	0.871	0.848
AirMLP8	300	0.801	0.775	0.751	0.848	0.805	0.773	0.878	0.830	0.812
	500	0.840	0.807	0.792	0.884	0.848	0.809	0.901	0.862	0.842
	700	0.885	0.848	0.807	0.896	0.876	0.826	**0.905**	0.883	0.859
AirMLP7h	300	0.797	0.768	0.738	0.836	0.876	0.826	0.856	0.825	0.805
	500	0.833	0.789	0.778	0.872	0.833	0.808	0.887	0.853	0.812
	700	0.848	0.823	0.796	0.883	0.855	0.829	**0.910**	0.860	0.844
AirMLP8h	300	0.808	0.760	0.769	0.853	0.819	0.785	0.876	0.836	0.807
	500	0.851	0.822	0.803	0.873	0.836	0.808	0.887	0.859	0.841
	700	0.867	0.832	0.810	0.887	0.867	0.840	**0.916**	0.867	0.848

**Table 5:** Performance with a batch size of 64, and a *loopback* of 20, increasing the number of neurons per layer.

Model	Neurons	R^2^
AirMLP6	900	0.901
	1100	0.912
	1500	0.926
AirMLP7	900	0.919
	1100	0.926
	1500	**0.932**
AirMLP8	900	0.917
	1100	0.928
	1500	0.925
AirMLP7h	900	0.915
	1100	0.921
	1500	0.927
AirMLP8h	900	0.917
	1100	0.921
	1500	0.923

## Data Availability

The source code and dataset can be accessed on Zenodo via the following links: [66,67].
